# Phytoplankton variability in relation to some environmental factors in the eastern coast of Suez Gulf, Egypt

**DOI:** 10.1007/s10661-015-4874-y

**Published:** 2015-09-26

**Authors:** Mohamed Z. Nassar, Nihal G. Shams El-Din, Samiha M. Gharib

**Affiliations:** National Institute of Oceanography and Fisheries, B.O.182, Suez, Egypt; National Institute of Oceanography and Fisheries, Alexandria, Egypt

**Keywords:** Phytoplankton, Diversity, Physicochemical parameters, Eastern Suez Gulf, Egypt

## Abstract

Water samples were seasonally collected from 12 stations of the eastern coast of Suez Gulf during autumn of 2012 and winter, spring, and summer of 2013 in order to investigate phytoplankton community structure in relation to some physicochemical parameters. The study area harbored a diversified phytoplankton community (138 species), belonging to 67 genera. Four algal groups were represented and classified as Bacillariophyceae (90 species), Dinophyceae (28 species), Cyanophyceae (16 species), and Chlorophyceae (4 species). The results indicated a relative high occurrence of some species namely.; *Pleurotaenium trabecula* of green algae; *Chaetoceros lorenzianus*, *Proboscia alata* var. *gracillima*, *Pseudosolenia calcar-avis*, and *Pseudo-nitzschia pungens* of diatoms; *Trichodesmium erythraeum* and *Pseudoanabaena limnetica* of cyanophytes. Most of other algal species were fairly distributed at the selected stations of the study area. The total abundance of phytoplankton was relatively low (average of 2989 unit/L) in the eastern coast of Suez Gulf, as compared its western coast and the northern part of the Red Sea. The diversity of phytoplankton species was relatively high (2.35–3.82 nats) with an annual average of 3.22 nats in the present study. The results concluded that most of eastern coast of Suez Gulf is still healthy, relatively unpolluted, and oligotrophic area, which is clearly achieved by the low values of dissolved phosphate (0.025–0.3 μM), nitrate (0.18–1.26 μM), and dissolved ammonium (0.81–5.36 μM). Even if the occurrence of potentially harmful algae species was low, the study area should be monitored continuously. The dissolved oxygen ranged between 1.77 and 8.41 mg/L and pH values between 7.6 and 8.41. The multiple regression analysis showed that the dissolved nitrate and pH values were the most effective factors that controlled the seasonal fluctuations of phytoplankton along the eastern coast of Suez Gulf during 2012–2013.

## Introduction

The northern end of the Red Sea bifurcates into the Sinai Peninsula, creating the Gulf of Suez in the west and the Gulf of Aqaba to the east. The gulf is relatively shallow and formed within a relatively young but now inactive Gulf of Suez Rift basin, dating back about 28 million years. It stretches some 300 km north by northwest, terminating at the Egyptian city of Suez and the entrance to the Suez Canal. Along the mid-line of the gulf is the boundary between Africa and Asia. The length of the gulf, from its mouth at the Strait of Jubal to its head at the city of Suez, is 314 km, and it varies in width from 19 to 32 km. The Gulf of Suez is relatively shallow, with a maximum depth of about 64 m; outside its mouth, the depth drops sharply to about 1255 m.

However, the Suez Gulf is subjected to sources of pollution such as shipping activities, where transport of oil continues to play a critical role in marine pollution in the northern part of the gulf and Suez Canal. On the other hand, extensive oil production operations are taking place in the gulf, both inshore and offshore. In addition, the gulf is subjected to industrial, agricultural, and domestic sewage; thermal pollution from power, desalination plants; and tourism activities (TEAM 2000, and NPA 2003a, b, c). In fact, there is no analysis of the tourism-related literature or recent analysis of impacts. The most published topics relate to coral breakage and its management. A full account of tourism’s environmental impacts is constrained by limited tourism data (Gladstone et al. 2013). However, the western coast of the gulf is considered more polluted than the eastern coast due to urbanization resulting from the population expansion, establishment of new industries along the coast such as fertilizer and cement factories, chemicals, and organic wastes from food processing factories at Suez City, and, in addition, to more tourism activities due to the establishment of numerous touristy villages. Thus, the Gulf of Suez could be fairly considered the most polluted area in the Red Sea (TEAM 2000 and NPA 2003a, b, c).

Noticeably, all these pollutants affected the marine ecosystem, which becomes under variable pressure, causing radical changes in marine organisms, including coral reefs, invertebrates, seagrasses, seaweeds, phytoplankton, and others (TEAM 2000 and NPA 2003a, b, c).

In fact, phytoplankton communities are the basis of many marine and freshwater food webs. Their composition fluctuates depending on hydrological conditions, such as light, temperature, salinity, pH, nutrients, and turbulence (Huertas et al. 2011). Typically, diatoms dominate coastal marine communities. However, other groups of phytoplankton can dominate depending on the combination of hydrological conditions and climatic variability (Leterme et al. 2006). Changes in dominant base groups/species often propagate up the food chain, impacting on fish, marine mammals, and birds (Donnelly et al. 2007). Phytoplankton are known to exhibit rapid responses to changes in environmental conditions and are therefore commonly acknowledged as excellent bioindicators of the impact of natural and seasonal changes in coastal ecosystems (Rimet and Bouchez 2012). Their susceptibility to environmental change is usually expressed by morphological and/or behavioral changes as well as by persistent or seasonally a typical differences in abundance and distribution (Leterme et al. 2010, 2013). Where mono-or class-specific blooms are observed on an annual basis, they often vary significantly in magnitude and/or duration between years (Ji et al. 2006 and Leterme et al. 2014).

The phytoplankton community structure in the northern part of the Red Sea was investigated by several workers and revealed variable biodiversity and community structure according to different ecological conditions and different spatial and temporal scales. Nassar (1994) recorded 76 species including 50 diatoms, 18 dinoflagellates, five blue-green algae, and three species of green algae in Suez Bay of the northern part of Suez Gulf. El-Sherif and Abo El-Ezz (2000) examined the distribution of plankton at Taba, Sharm El-Sheikh, Hurgada, and Safaga at northern Red Sea, recording 41 diatom species, 53 dinoflagellates, 10 cyanophytes, and two chlorophytes. Deyab et al. (2004) recorded 200 phytoplankton species along the Suez Canal, Suez Gulf, and the northern part of the Red Sea with clear dominance of diatoms. Shams El-Din et al. (2005) identified 110 phytoplankton species belonging to seven classes on both sides of the Suez Gulf. Nassar (2007a) studied the phytoplankton dynamics in the western coast of Suez Gulf and recorded 144 species of different groups, and Nassar (2007b) conducted similar study on the phytoplankton abundance in the coastal waters of the Aqaba Gulf, recording 127 taxa. Also, Al-Najjar et al. (2007) studied the seasonal dynamics of phytoplankton in the Gulf of Aqaba. Madkour et al. (2010) reported that the spatial distribution of phytoplankton showed that Gulf of Suez differs in the dominant species and timing of abundance from both Gulf of Aqaba and the southern sites of Sinai Peninsula. Recently, a checklist of 207 phytoplankton species is detected in the Egyptian waters of the Red Sea and some surrounding habitats during the period 1990–2010 (Nassar and Khairy 2014). In fact, the available literatures on phytoplankton population dynamic in the eastern coast of the gulf are scarce, and information is lacking concerning phytoplankton in this area.

The aim of the present work is to follow up the changes that might take place in the standing crop and community structure of the phytoplankton in the coastal waters of eastern coast of the Suez Gulf in response to changes in the physicochemical characters of water and to compare the results with the previous studies of the surrounding habitats.

## Materials and methods

### Description of sampling stations

**Fig. 1:**
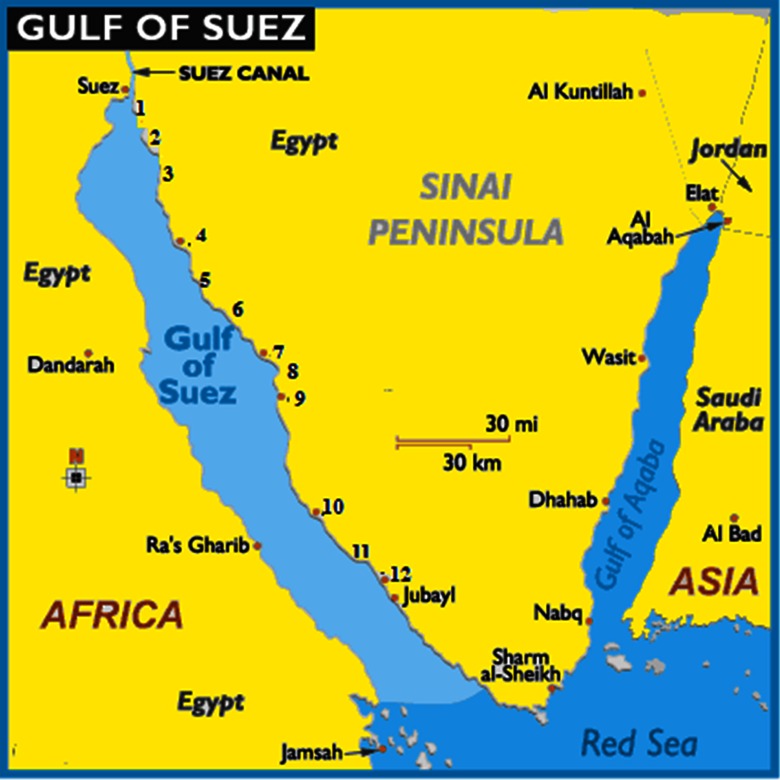
Positions of the sampling stations (1–12)

Twelve stations were selected along the eastern coast of the Suez Gulf as shown in Fig. 1. These stations are subjected to different ecological conditions due to the touristic and human activities, sewage and oil effluents, and industrial and thermal effects: St. 1 is located near the Electrical Power Station of Ayon Mousa at about 500 m of the coast and is subjected to thermal water discharge. St. 2 is situated near a tourist village namely Tamara Crouze with low human and tourist activities. St. 3 is located near the beach of Ras Sedr and is subjected to high tourist and human activities especially during summer months. St. 4 is located near a new tourist village namely Daghish village, at which low human and tourist activities were observed. St. 5 is a sandy beach located north of the Hammam Pharaon and is relatively far from the human activities. St. 6 is located near the Hammam Pharaon hot springs; its water is relatively hot and characterized by high vegetations and bad odors. St. 7 (Abu-Zenima) is situated at about 2.5 km from Abu-Zenima City. St. 8 is located near from the manganese factory of Abu-Zenima and is subjected to Mn effluents of this factory. St. 9 (Abu-Redis) is located in the south of Abu-Redis City and is subjected to sewage and oil effluents discharged from the oil charging and discharging company. St. 10 (Petropil) is situated near the Petropil Oil Company and is subjected also to oil effluents. St. 11 (Al-Konaysa) is located near a fishing harbor of the Fisheries Commission and is subjected to fishing and human activities. St. 12 (Al-Tur) is situated near the eastern side of Al-Tur fishing harbor and is also subjected to fishing and human activities.

### Phytoplankton estimations

Water samples were seasonally collected using Ruttiner bottles from the sub-surface waters of different 12 stations during autumn of 2012 (November) and winter (January), spring (April), and summer (August) of 2013 (Fig. 1). Cell abundance and composition of phytoplankton were estimated according to sedimentation method (Utermöhl 1958). The species identification was carried out following Peragallo and Peragallo (1908), Ghazzawi (1939), Cupp (1943), Prescott (1962), Bourrelly (1968), Ferguson (1968), Sournia (1986), Mizuno (1990), and Al-Kandari et al. (2009). Then, the phytoplankton species are updated according to the taxonomic database sites, like algaebase.com (ab), World Register of Marine Species (WoRMS), Canadian Register of Marine Species (CaRMS), Nordic Microalgae and Aquatic Protozoa (NOD), and Integrated Taxonomic Information System (ITIS).

### Physicochemical parameters

Water temperature was measured by using a simple pocket thermometer graduated to 0.1 °C. The pH value of water samples was measured in situ using a pocket pH meter model Orion 210. Dissolved oxygen was fixed in field and measured according to the modified Winkler’s method according to (Strickland and Parsons 1972), and the dissolved inorganic nutrients (NO_3_, NH_4_ and PO_4_) were determined spectrophotometrically, and the results were expressed in micromolar according to the methods described by APHA (2005).

### Statistical analysis

The correlation matrices was applied to total phytoplankton counts, phytoplankton classes, dominant species, and the physicochemical parameters at confidence limit 95 % and *n* = 47. Multiple regression was calculated for phytoplankton during each season, using the program of STATISTICA Version 5. Similarity index between the stations of the study area, based on phytoplankton community structure, was calculated, using the program of Primer 5. The species diversity (H′) was calculated according to Shannon and Weaver (1963).

## Results

### Physicochemical parameters

The results of physicochemical parameters of seawater samples collected from the eastern coast of Suez Gulf during 2012–2013 are shown in Table 1.

**Table 1:** Seasonal variations of temperature (°C), pH value, DO (mg O_2_/L), and the nutrients PO_4_, No_3_, and NH_4_ (μM) along the eastern coast of Suez Gulf during 2012–2013

Autumn 2012													
Station	1	2	3	4	5	6	7	8	9	10	11	12	Average
Temp	22.00	23.00	22.40	22.70	23.50	22.80	23.80	23.10	22.60	24.00	23.20	22.20	22.94
pH	8.22	8.00	8.20	8.00	8.00	8.10	8.05	8.12	7.66	7.70	7.80	7.80	–
DO	3.00	3.63	2.38	4.25	3.56	3.30	3.38	3.11	7.50	6.50	4.60	4.32	4.12
PO_4_	0.122	0.166	0.09	0.173	0.147	0.134	0.144	0.126	0.30	0.26	0.186	0.175	0.17
NO_3_	0.55	0.70	0.52	0.71	0.61	0.50	0.61	0.40	1.26	1.10	0.77	0.73	0.71
NH_4_	2.66	1.28	2.32	1.26	1.16	1.53	1.51	1.63	0.84	1.07	1.10	1.17	1.46
Winter 2013													
Temp	17.00	18.40	17.50	18.00	18.8	18.2	19.00	18.6	17.70	19.00	18.50	17.30	18.16
pH	8.30	7.90	8.22	7.90	8.00	8.10	8.10	8.20	7.60	7.85	7.90	7.90	–
DO	3.32	4.14	2.88	4.26	4.1	3.78	3.90	3.40	8.41	7.51	5.27	4.63	4.63
PO_4_	0.11	0.14	0.09	0.14	0.13	0.13	0.11	0.11	0.28	0.25	0.17	0.15	0.15
NO_3_	0.32	0.46	0.37	0.47	0.45	0.42	0.43	0.38	0.93	0.89	0.60	0.52	0.52
NH_4_	4.72	2.28	4.21	2.18	2.32	2.60	2.40	2.96	1.62	1.86	1.90	2.12	2.60
Spring 2013													
Temp	25.00	26.80	25.50	26.20	27.40	26.40	27.00	27.00	26.00	ND	27.20	25.20	26.20
pH	8.32	8.00	8.30	7.90	8.00	8.20	8.17	8.22	7.80	ND	7.83	7.87	–
DO	1.83	3.43	1.77	3.72	3.4	2.74	3.32	2.37	5.25	ND	4.75	4.1	4.425
PO_4_	0.04	0.08	0.04	0.08	0.07	0.06	0.07	0.05	0.12	ND	0.11	0.09	0.10
NO_3_	0.25	0.50	0.26	0.53	0.48	0.4	0.47	0.34	0.75	ND	0.68	0.6	0.64
NH_4_	0.81	1.52	2.18	1.26	1.56	1.71	1.58	1.87	2.40	ND	0.84	1.1	0.97
Summer 2013													
Temp	28.1	30.6	28.7	29.4	31.4	30	31.5	31	29.4	31.2	31.2	28.5	30.1
pH	8.41	8.3	8.4	8.3	8.3	8.35	8.33	8.4	8.3	8.12	8.14	8.26	–
DO	3.1	4.45	2.34	4.53	4.14	3.32	3.42	3.26	4.53	5.37	5.22	4.74	4.03
PO_4_	0.03	0.05	0.02	0.05	0.04	0.04	0.04	0.03	0.05	0.06	0.06	0.05	0.04
NO_3_	0.21	0.34	0.18	0.35	0.26	0.25	0.26	0.24	0.35	0.42	0.4	0.36	0.30
NH_4_	2.55	2.62	4.11	2.55	3.4	3.62	3.46	4.0	5.36	2.1	2.37	2.5	3.22

*ND* not measured

The temperature was typical of the north part of the Suez Gulf, ranging from a minimum of 17.00 °C during winter at St. 1 and a maximum of 31.50 °C during summer at St. 7 with an annual mean value of 24.35 °C. The normal thermal cycle was clear in the study area, showing the highest temperature during summer (30.10 °C), while in winter, the lowest ones were reached (18.16 °C).

Seawater pH lied in the alkaline side during all seasons with almost the highest values during summer. The lowest value of pH was recorded during winter at St. 9 (7.60) and the highest during summer at St. 1 (8.41). Whereas, seawater dissolved oxygen (DO) varied between a minimum of 1.77 mg/L during spring at St. 3 and a maximum of 8.41 mg/L during winter at St. 9 with small seasonal differences and an annual average of 4.30 mg O_2_/L.

As far as nutrients are concerned, the reactive phosphate (PO_4_) was very low during spring and summer at all stations, whereas the maximum value was recorded during autumn at St. 9 (0.30 μM). The dissolved nitrate (NO_3_) in the gulf ranged between a maximum value of 1.26 μM during autumn at St. 9 and a minimum of 0.18 μM during summer at St. 3. The dissolved ammonium (NH_4_) varied between a minimum of 0.81 μM during spring at St. 1 and a maximum of 5.36 μM during summer at St. 9, which may be due to the effect of sewage and oil effluents. Generally, nitrate and phosphate exhibited a seasonal cycle with lower concentrations during summer, while dissolved ammonium was the most abundant source of nitrogen during summer (Table 1).

### Phytoplankton

#### Community composition

The study area showed a discrete phytoplankton diversity (138 species), belonging to 67 genera. Four algal groups were represented in the eastern coast of Suez Gulf belonging to Bacillariophyceae (90 species), Dinophyceae (28 species), Cyanophyceae (16 species), and Chlorophyceae (4 species) (Table 2).

**Table 2:** Relative counts of the recorded phytoplankton species (unit/L) along the eastern coast of Suez Gulf during 2012–2013

Diatoms	1	2	3	4	5	6	7	8	9	10	11	12
*Amphiprora alata* (Ehrenberg) Kützing (ab)	+		+	+	+		+		+	+		
Amphiprora paludosa W. Smith (ab)	+	+	+	+	+	+	+	+	+	+	+	+
*Amphora grevilleana* Gregory (WoRMS)	+											
*Amphora lineolata* Ehrenberg (ab)		+	+		+	+	+	+	+	+	+	
*Amphora marina* W. Smith (ab)		+	+	+	+	+	+	+	+	+	+	+
*Amphora ovalis* (Kützing) Kützing (WoRMS)									+			
*Asterionella* sp.	+		+									
*Asterolampra* sp.				+								
*Aulacoseira granulata var angustissima* (O.F.Müller) Simonsen (ab)	+	+	+	+	+	+	+	+	+	+	+	+
*Aulacoseira italica* (Ehrenberg) Simonsen (ab)	+	+	+							+	+	+
*Bacillaria paradoxa* J.F. Gmelin (ab)									+			+
*Campylodiscus hibernicus* Ehrenberg (ab)	++	+	+	+	+	–	+	+	+	+	+	+
Chaetoceros coarctatus* Lauder (ab)		+		+				+		+	+	-
Chaetoceros curvisetus* Cleve (ab)		+										
Chaetoceros densus* (Cleve) Cleve (ab)									+		+	
Chaetoceros lorenzianus* Grunow (ab)	+	+	+	+++	++	++	+	+	++	+	+	+
Chaetoceros peruvianus* Brightwell (ab)				+					+			
*Climacodium biconcavum* Cleve (WoRMS)				+	+		+		+	++	++	
*Climacosphenia moniligera* Ehr. (ab)	+	++	+	+	++	++	++	++	++	+	++	++
*Cocconeis placentula* Ehrenberg (ab)		+			+	+	+	+	+	+	+	–
*Coscinodiscus centralis* Ehrenberg (WoRMS)		+						+				
*Coscinodiscus granii* Gough (ab)		+				+	+	+				
*Coscinodiscus marginatus* Ehrenberg (ab)		+	+									
*Coscinodiscus radiatus* Ehrenberg (WoRMS)		+++	–	+	+	+	+	+	+	+	–	–
Cyclotella meneghiana* Kützing (ab)				+		+						
*Cymbella aspera* Ehrenberg Cleve (WoRMS)									+			
*Cymbella sp.*	+	+	+	+	+				+			+
*Cymbella ventricosa* (C.Agardh) C.Agardh (ab)			+	+	+	+	+		+			+
*Diploneis interrupta* (Kützing) Cleve (ab)				+					++			
*Diploneis sp.*				+	+		+		+			
*Fragillaria pectinalis* (O.F.Müller), Lyngbye (ab)							+					
*Fragillaria construens* Ehrenberg Grunow (ab)							+					
*Fragillaria sp.*	+	+	+	+				+	+			+
*Gramatophora marina* (Lyngbye) Kützing (ab)		+		+	+	+	+	+	+		+	
*Gramatophora oceanica Ehrenberg* (WoRMS)				+		+			+			
*Guinardia flaccida* (Castracane) H. Peragallo (ab)	+	+++	+	+++	++	++	++	++	+++	++	++	+
*Gyrosigma acuminatum* (Kützing) Rabenhorst (WoRMS)	+	+	+	+	+	+		+	++		+	+
*Gyrosigma attenuatum* (Kützing) Rabenhorst (ab)	+++	++	++	++	++	++	++	++	+++	+++	++	+++
*Gyrosigma balticum* (Ehrenberg) Rabenhorst (WoRMS)	+	+	+	+		+			+			+
*Hemiaulus membranaceus* Cleve				+		+			+			
*Hemiaulus sinensis Greville*									+			
*Lauderia* annulata Cleve (WoRMS)											+	
*Leptocylindrus danicus* (ab)		+		+	+	+	+	+		+++	+	
*Leptocylindrus minimus* Granv (WoRMS)	+++	+++	+	+	++	+	++	++	+	+	+	+
*Leptocylindrus sp.*	+		+	+					+			+
*Licmophora abbreviata* C. Agardh (ab)				+					+			
*Licmophora flabellata* C. Agardh (ab)	+	+	++	++	+	++	+	++	++	++	+	++
*Licmophora gracilis* (Ehrenberg) Grunow (WoRMS)	+	+	+	+	+	+	+	+	+	+	+	+
*Mastogloia sp.*		+					+	+			+	
*Melosira* sp.				+								+
*Melosira varians* C. Agardh (ab)	+							+		+	+	
*Navicula dicephala* (Ehrenberg) W. Smith (ab)			+	+			+		+			+
*Navicula tripunctata* (O.F. Müller) Bory de Saint-Vincent (WoRMS)	+	+	+	++	+	+	+	+	+	+	+	+
*Navicula placentula* (Ehrenberg) Kützing (ab)				+	+	+	+		++	+	+	
*Navicula* sp.			+	+		+	+		+			+
Nitzschia acicularis (Kütz.) W.Sm.	+	+	+	+	+	+	+	+	++	+	+	+
Nitzschia closterium* (Ehrenberg) W. Smith (ab)	+		+	+					+			+
Nitzschia longissima* Brébisson) Ralfs in Pritchard (ab)	++	+	+	+	++	+	+	++	++	++	+++	+
*Nitzschia obtusa* W. Smith (ab)	+	+	+	+	+	+	+	+	+	+	+	+
Nitzschia pungens* var. atlantica (Grunow ex Cleve) G.R.Hasle, (ab)	+	+	+	+++	++	++	+	++	+++	+	++	+
*Nitzschia sigma* (Kützing) W. Smith (ab)	+	+	+	++	+	++	++	+	++	++	+++	++
*Nitzschia vermicularis* (Kütz.) Hantzsch in Rabenh	+						+					
*Odontella aurita* (Lyngbye) C.A. Agardh (ab)		+		+	+	+	+	+	+			
*Odontella obtusa* (Kützing) Ralfs (ab)	+	++	+	+	+	+	+	++	+	+	++	++
*Odontella sinensis* (Greville) Grunow ( WoRMS)									+			
*Paralia sulcata* (Ehrenberg) Cleve (ab				+								
*Plagiotropis lepidoptera* (Gregory) Kuntze (ab)		+		+	+	+	+	+	++	+	+	
*Pleurosigma angulatum* W. Smith (ab)		+			+	+	+	+	+	+	+	
Proboscia alata var.gracillima* (Brightwell) Sündstrom (ab)	++	+++	++	+++	+++	++	+++	+++	+++	++	++	+++
Proboscia alata form indica* Brightwell) Sündstrom (ab)		+	+	++					+			
*Pseudosolenia bergoni* H. Péragallo (ab)									+			
*Pseudosolenia calcar avis* (Schultze) Sundström (ab)	++	+++	++	+++	+++	++	++	+++	+++	+++	+++	++
*Rhizosolenia fragilissima* Bergon		+		+	+	+	+				+	
*Rhizosolenia imbricata* (Cleve) Schröder WoRMS)		++++	+	+	+	+	+	+	+++		++	
*Rhizosolenia stoterfothii* H. Peragallo (ab)		+		+	+	+	+			+	+	
*Rhizosolenia styliformis* Brightwell (ab)	+	+		+	+				+	+	+	+
Skeletonema costatum* (Greville) Cleve (ab)	+	+	+	+		+			+			+
*Stephanopyxis turis* (Greville & Arnott in Gregory) Ralfs in Pritchard		+						+	-	+	+	
*Striatella unipunctata* (Lyngbye) C. Agardh (ab)				+					+			
*Surirella minuta* Brébisson (WoRMS)	++	+	++	+	+	+	+	+	+	+	+	+
*Surirella robusta* Ehrenberg (ab)	+		+									+
*Synedra acus* Kütz.	+	+	+	+		+	+		+			+
*Synedra crystalline* (C.Agardh) Kützing (WoRMS)		+										
*Synedra ulna* (Nitzsch) Ehrenberg (ab)	++	++	+	++	++	++	++	++	++	++	+++	++
*Synedra undulata* (J.W.Bailey) Gregory (ab)	+	+	+		+	+	+	+	+	+	+	+
Thalassionema nitzschioides* (Grunow) Mereschkowsky (ab)	+	+	+	+	+			+	+		+	+
*Thalassiosira* sp.	+				+	++	++					
*Thalassiothrix frauenfeldii* (Grunow) Grunow (WoRMS)		++		++	+	+		+	++	+	+	
*Thalassiothrix longissima* Cleve & Grunow (ab)	++	++	++	+++	++	++	++	++	+++	+++	+++	+++
*Trachyneis aspera* (Ehrenberg; Ehrenberg) Cleve		+		+	++	+	+	+		+	++	
Cyanophytes												
*Anabaena* sp.		+										
*Chroococcus minutus* (Kütz.) Nägeli	+		++		++		+	+	+		+	+
*Chroococcus turigidus* (Kützing) Nägeli (ab		+	+		+				+			+
*Coelosphaerium* sp.	+		+	+	+	+			+		+	+
*Lyngbya major* Meneghini ex Gomont (ab)								+		++	+	
*Lyngbya majuscula* Harvey ex Gomont (WoRMS)		+			+		+			++	+	
*Microcystis* sp.		+	+	+	+				+		+	+
Trichodesmium erythraeum *(Ehrenberg) Geitler (ab)	+	+	+	+	+++	+	+++	++	++	++++	+++	+++
Pseudanabaena limnetica* (Lemmermann) Komárek (ab)	++	++	++	+	+++	++	+++	++	++	+++	++	++
Oscillatoria simplicissima* Gomont (WoRMS)		+	+	+	+	+	+	++	+	+++	+	++
*Oscillatoria* sp.	+	+++	++	+	++	+	++	+	++	++	++	++
Oscillatoria tenuis* C. Agardh (WoRMS)		+	+		+		+	+	+	++	+	+
*Phormidium* sp.	++	++	++	+	++	++	++	++	++	+++	++	++
Planktothrix formosa*	+	+	+	+	+	+	+	+	+	++	+	++
*Spirulina major* Kützing ex Gomont (WoRMS)	++											+
*Spirulina* sp.			+							+		
Dinoflagellates												
*Ceratium breve* (Ostenfeld & Schmidt) Schroder (ab)	+		+							+	+	+
*Ceratium egyptiacum* Halim (ab)									+			
*Ceratium extensum* (Gourret) Cleve-Euler (ab)	+						+					+
Ceratium furca* (Ehrenberg) Claparède & Lachmann (WoRMS)	+	+	+	+				+	+			+
Ceratium fusus* (Ehrenberg) Dujardin (ab)	+		+		+				+			+
*Ceratium karastenii* Pavillard (WoRMS)									+			
*Ceratium macroceros* (Kofoid) Peters (ab)	+	+	+		+			+	+		+	+
*Ceratium massiliense* (Gourret) E.G.Jørgensen (ab)	+	+	+	+		+	+	+	++		+	+
*Ceratium trichoceros* (Ehrenberg) Kofoid (WoRMS)	+	+	+	++	+	+	+	+	++	+++	+++	+
Ceratium tripos* (O.F.Müller) Nitzsch (WoRMS)	+	+	+	+	+	+	+	+	+	++	++	+
Dinophysis caudata* Saville-Kent (ab)		+							+	+	+	
*Dinophysis sp.*				+								
*Diplopsalis lenticula*						+			+		+	
*Exuviaella compressa* (Bailey) Knudsen & in Ostenfeld (ab)		+		+	+	++	+	+	+	++	++	
*Goniaulax* sp.					+		+			+		
*Gymnodinium* sp.				+					+			
*Oxytoxum gracile* J.Schiller						+					+	
*Phalacroma* sp.				+								
*Podolampas palmipes* Stein		+				+		+				
*Prorocentrum compressum* (J.W. Bailey) Abé ex Dodge									+			
Prorocentrum micans* Ehrenberg (ab)		+		+								
Prorocentrum minimum* (Pavillard) Schiller		+					+		+	+		
*Protoperidinium minutum* (Kofoid) Loeblich III		+		+	+	+	+	+	+	+	+	
*Protoperidinium cerasus* (Paulsen) Balech (ab)	+	+	+	+	+;	+	+	+	+	+	+	+
*Protoperidinium depressum* (Bailey) Balech (CaRMS)		+				+		+		+		
*Protoperidinium divergens* (Ehrenberg) Balech (CaRMS)								+				
*Protoperidinium ovatum* Pouchet (CaRMS)				+		+						
*Pyrophacus horologicum* Stein (ab)		+							+			
Chlorophytes												
*Dictyosphaerium* sp.		+									+	
*Pediastrum clathratum.* (Schröder) Lemmermann (ab)											+	
*Pleurotaenium trabeculum* Nägeli (ab)	+++	+	+++	+++	++++	+++	++++	+++	+++	+++	+++	+++
*Treubaria crassipina* G. M. Smith (WoRMS)*4*							+	+		++		0

0–50 rare +, >50–100 frequent ++, >100–200 common +++, >200 abundant ++++

The species marked with asterisks are potential harmful

The diatoms were the most dominated group, forming about 67.00 % of the total counts of phytoplankton, followed by Cyanophytes that represented about 17.00 % of the total abundance. On the other hand, Dinophyceae and Chlorophyceae formed collectively about 15.50 % of the total counts of phytoplankton (Table 3).

**Table 3:** Seasonal variations of phytoplankton counts (unit/L) at the different stations along the eastern coast of Suez Gulf

Autumn 2012														
Station	1	2	3	4	5	6	7	8	9	10	11	12	Average	Percentage
Algal group														
Diatoms	1790	1980	1286	3185	2598	2051	2035	2374	2703	3738	3696	2045	2457	65 %
Dinoflagellates	233	278	167	328	50	217	67	100	980	167	484	278	279	7 %
Cyanophytes	278	362	457	300	756	328	678	551	317	2355	1167	561	676	18 %
Chlorophytes	378	200	233	233	400	333	450	250	250	633	311	1000	389	10 %
Total	2679	2820	2143	4046	3804	2929	3230	3275	4250	6893	5658	3884	3801	100 %
Winter 2013														
Diatoms	1657	1970	2319	2748	2481	2280	1967	1870	3997	1943	2002	3917	2429	82 %
Dinoflagellates	200	295	78	228	228	328	451	195	384	401	134	250	264	9 %
Cyanophytes	0	178	379	0	601	67	100	67	467	311	67	1111	279	9 %
Chlorophytes	0	0	0	0	0	0	0	0	0	0	0	0	0	0 %
Total	1857	2443	2776	2976	3310	2675	2518	2132	4848	2655	2203	5278	2973	100 %
Spring 2013														
Diatoms	1002	2004	1364	3042	1374	2265	1947	1601	3393	Nd	1881	841	1883	69 %
Dinoflagellates	117	128	100	357	0	167	0	145	134	ND	145	100	127	5 %
Cyanophytes	311	367	345	245	751	473	667	578	478	ND	818	324	487	18 %
Chlorophytes	150	278	200	100	178	100	133	356	333	ND	378	178	217	8 %
	1580	2777	2009	3744	2303	3005	2747	2680	4338	ND	3222	1443	2713	100 %
Summer 2013														
Diatoms	1635	2285	929	1112	1108	857	967	1302	1835	845	1547	1290	1309	53.03 %
Dinoflagellates	167	506	167	234	312	234	117	251	596	117	829	195	310	12.57 %
Cyanophytes	754	1161	748	412	756	306	562	333	678	345	533	440	586	23.72 %
Chlorophytes	200	78	233	78	378	100	1000	100	133	666	100	100	264	10.68 %
Total	2756	4030	2077	1836	2554	1497	2646	1986	3242	1973	3009	2025	2469	100 %
Total average														
Diatoms	1521	2060	1475	2522	1890	1863	1729	1787	2982	2175	2282	2023	2026	67.48 %
Dinoflagellates	179	302	128	287	148	237	159	173	524	228	398	206	247	8.24 %
Cyanophytes	336	517	354	239	716	294	502	382	485	1004	646	609	507	16.89 %
Chlorophytes	182	139	167	103	239	133	396	177	179	433	197	320	222	7.39 %
Total	2218	3018	2123	3151	2993	2527	2785	2518	4170	3840	3523	3158	3002	100 %

*Note: ND means not measured

The phytoplankton diversity displayed wide spatial variations. The station 9 was reported as the most diversified community (93 species), while station 1 recorded the lowest diversified one (59 species) (Table 2). On the other hand, there were no distinct seasonal variations in phytoplankton diversity, where the three seasons: autumn, winter, and spring harbored closed number of species (64, 66, and 69 species), respectively, whereas the summer harbored relatively low number (53 species).

Regardless of the large number of phytoplankton species in the study area, only nine species were perennial (occurring during the four seasons). These species are *Guinardia flaccida*, *Gyrosigma attenuatum*, *Nitzschia longissima*, *Nitzschia sigma*, *Odontella obtusa*, *Synedra ulna*, *Thalassiothrix longissima*, *Phormidium* sp., and *Ceratium trichoceros*. Whereas, 21 species appeared during the three seasons and are considered as semi-perennial. The rest number of species was observed either for one or two seasons. On the other hand, there were few species restricted to one station such as *Amphora grevilleana* (St. 1); *Chaetoceros curvisetus*, *Synedra crystallina*, and *Anabaena* sp. (St. 2); *Asterolampra* sp., *Paralia sulcata*, *Dinophysis* sp., *and Phalacroma* sp. (St. 4); *Fragillaria pectinalis* and *Fragillaria construens* (St. 7); *Protoperidinium divergens* (St. 8); *Amphora ovalis*, *Cymbella aspera*, *Hemiaulus sinensis*, *Odontella sinensis*, *Rhizosolenia bergoni*, *Ceratium egyptiacum*, *Ceratium karastenii*, and *Prorocentrum compressum* (St. 9), whereas *Pediastum clathratum* appeared only at St. 11 (Table 2).

#### Seasonal and spatial variations of phytoplankton community

**Fig. 2:**
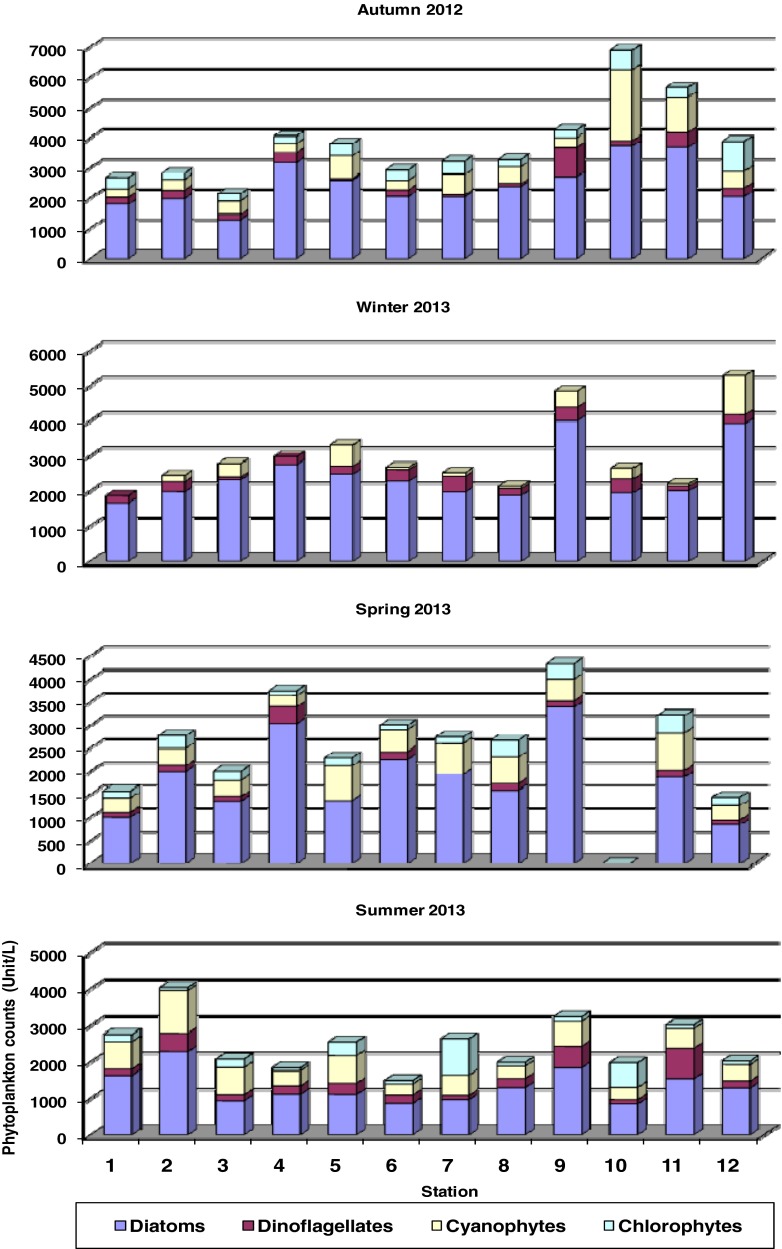
Seasonal variations of phytoplankton abundance (unit/L) at the different stations in the eastern coast of Suez Gulf during 2012–2013

The diatoms prevailed during all seasons forming numerically the highest percentage (82 %) during winter, followed by cyanophytes, which formed the highest percentage during summer (24 %). The chlorophytes were absent during winter at all stations and formed almost equal percentages during the three other seasons. The fourth group, dinoflagellates, formed numerically (5–13 %) of the total count during the study period. Like the seasonal pattern, the spatial distribution of phytoplankton revealed the dominance of diatoms at all stations (Table 3), forming numerically from 57 % (St. 10) to 80 % (St. 4). The cyanophytes and chlorophytes showed the inverse pattern, recording the highest percentage (26 %, St. 10) and the lowest (8 %, St. 4) for the former and (11 %, St. 10; 3 %, St. 4) for the later. The dinoflagellates displayed the highest contribution at St. 9 (13 %) and the lowest one at St. 5 (5 %) as shown in Fig. 2.

#### Standing crop

The total abundance of phytoplankton was relatively low in the present study of the eastern coast of Suez Gulf (average of 2989 unit/L) as compared with the previous studies (Table 4). The total phytoplankton showed the highest counts during autumn 2012 with an average of 3801 unit/L, followed by winter (average of 2973 unit/L), spring (average of 2713 unit/L), and summer (average of 2469 unit/L) as shown in Table 3 and Fig. 2. The peak of autumn was due to the co-dominance of *C. lorenzianus* (4.24 %), *G. attenuatum* (6.16 %), *P. pungens* (4.05 %), *P. alata* var. *gracillima* (4.37 %), *P. calcar-avis* (7.35 %), *T. erythraeum* (3.98 %), *Pseudanabaena limnetica* (3.75 %), and *P. trabecula* (9.41 %). However, some of these species were also observed with relative high counts during summer and spring, 2013 such as *P. alata* var. *gracillima*, *P. calcar-avis*, and *P. trabecula*. Most of other algal species were fairly distributed at the different stations in the coastal waters of eastern coast of Suez Gulf during 2012–2013.

**Table 4:** The number of species and abundance of phytoplankton in Suez Gulf

	Western coast of Suez Gulf, Nassar (2000)				Western coast of Suez Gulf, Nassar (2007a)				Eastern coast of Suez Gulf (present study)			
Algal group	G	spp	Total counts	%	G	spp	Total counts	%	G	spp	Total counts	%
Diatoms	28	47	4252	72.53	40	89	10958	70.30	42	90	2019	67.54
Dinoflagellates	9	18	1278	21.80	11	30	957	6.13	12	28	245	8.20
Cyanophytes	3	4	314	5.35	7	12	802	5.14	9	16	507	17.00
Chlorophytes	0	0	0	0.00	10	12	2869	18.4	4	4	218	7.29
Euglenophytes	0	0	0	0.00	1	1	5	0.03	0	0	0	0.00
Silicoflagellates	1	1	17	0.29	0	0	0	0.00	0	0	0	0.00
Total	41	70	5862	100	69	144	15591	100	67	138	2989	100

On the spatial scale, the average total count was relatively low in all stations. The relatively high abundance was recorded at St. 9 followed by St. 10 with total counts of 4170 and 3840, respectively, whereas St. 1 and St. 3 sustained the lowest average total counts (2218 and 2123 unit/L, respectively) (Table 3). However, St. 9 sustained relatively high counts of phytoplankton during the whole period, due to the co-dominance of *P. pungens* (3.06 %), *P. alata* var. *gracillima* (3.39 %), *P. calcar-avis* (3.80 %), and *P. trabecula* (4.29 %). The species *P. trabecula* appeared also at St. 10 (9.54 %), with the contribution of *Melosira granulata* (4.77 %), *T. longissima* (3.90 %), *T. erythraeum* (5.78 %), and *P. limnetica* (3.76 %).

#### Species diversity

The total average of diversity in the eastern coast of Suez Gulf was 3.22 (Table 5). The diversity of phytoplankton sustained a maximum of 3.82 during winter at St. 9, in which the highest numbers of phytoplankton species were observed (49 spp.). On the other hand, the minimum diversity of 2.35 was found during summer at St. 7, in which the lowest numbers of species were recorded (19 spp.). On the temporal scale, the winter season sustained relatively higher diversity (3.43), whereas the other three seasons sustained relatively closed diversities (Table 5). Spatially, St. 1 and 12 sustained the lowest average of diversity (2.97 and 3.03), respectively, against the highest diversity at St. 9 (3.47).

**Table 5:** Seasonal variations of species diversity (nats) at the different stations in the eastern coast of Suez Gulf

Station	1	2	3	4	5	6	7	8	9	10	11	12	Average
Autumn 2012	2.71	3.28	3.02	3.06	3.13	3.06	3.09	3.22	3.43	3.35	3.44	2.75	3.13
Winter 2013	3.06	3.46	3.28	3.61	3.52	3.42	3.61	3.26	3.82	3.6	3.15	3.33	3.43
Spring 2013	3.00	3.43	3.11	3.67	3.27	3.37	3.34	3.12	3.41	ND	3.48	2.86	3.28
Summer 2013	3.11	3.4	2.88	3.00	2.88	2.93	2.35	3.14	3.24	3.36	3.33	3.19	3.06
Average	2.97	3.39	3.07	3.33	3.20	3.19	3.09	3.18	3.47	3.43	3.35	3.03	3.22

## Statistical analysis

### Correlation matrices and multiple regressions

**Fig. 3:**
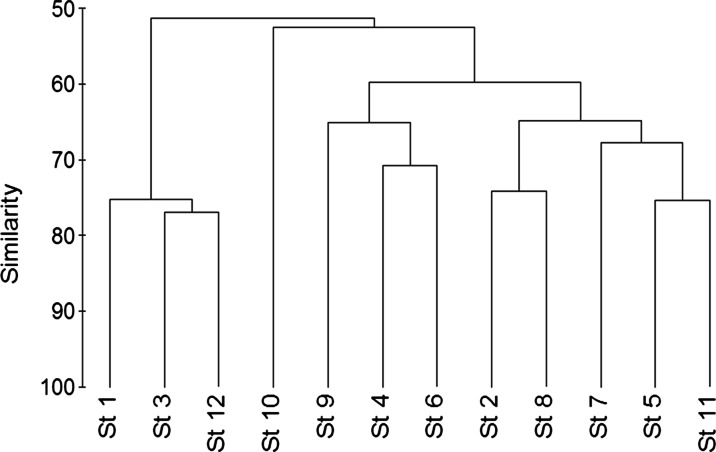
Bray-Curtis of similarity of phytoplankton abundance between the different stations

The statistical analysis of the data indicated that the phytoplankton abundance was positively correlated with nitrate (*r* = 0.66) and dissolved oxygen (*r* = 0.51) but inversely correlated with pH values (*r* = −0.63), whereas the groups and dominant species showed varied correlations with physicochemical characteristics as shown in Table 6. The multiple regression analysis indicated that the dissolved nitrate and pH values were the most effective factors that controlled the seasonal fluctuations of phytoplankton in the eastern coast of Suez Gulf during 2012–2013. The regression model was phytoplankton counts = 1379.2341 + 0.662 NO_3_ – 0.33 pH (MR = 0.662, *N* = 47, *p* < 0.1278). The similarity index revealed four clusters (Fig. 3).

**Table 6:** The correlations between total phytoplankton counts, phytoplankton classes, dominant species, and the physicochemical parameters during 2012–2013

	Temp	pH	DO	PO_4_	NO_3_	NH_4_
	°C	–	Mg O_2_/L	μM		
Total phytoplankton	−0.20	−**0.63**	**0.51**	0.44	**0.66**	−0.47
Diatoms	−**0.51**	−**0.69**	**0.48**	**0.69**	**0.63**	−**0.46**
Dinoflagellates	0.01	−0.22	**0.52**	0.33	0.34	−0.09
Cyanophytes	0.27	−0.18	0.19	0.14	0.27	−0.18
Chlorophytes	**0.38**	−0.08	−0.02	0.02	0.17	−0.22
*Chaetoceros lorenzianus*	−0.08	−0.35	0.07	**0.42**	**0.50**	−**0.51**
*Gyrosigma attenuatum*	0.09	−0.20	0.14	0.39	**0.49**	−0.27
*Pseudosolenia calcar*-*avis*	0.10	−0.24	−0.03	0.28	**0.40**	−**0.49**
*Pleurotaenium trabecula*	0.38	−0.02	−0.05	−0.03	0.11	−0.16
*Proboscia alata* var. *gracillima*	0.33	0.27	0.07	−0.10	−0.15	0.23
*Pseudo-nitzschia pungens*	−**0.45**	−**0.43**	0.31	**0.63**	**0.52**	−0.32
*Trichodesmium erythraeum*	0.29	−0.15	0.17	0.14	0.25	−0.17
*Pseudanabaena limnetica*	**0.50**	0.10	−0.01	−0.04	0.10	−0.03

Bold correlations are significant at *p* < 0.05 and *n* = 47)

## Discussion

Coastal waters are characterized by a high degree of spatial and temporal variability of environmental parameters (Bosak et al. 2012). These ecosystems face increasing anthropogenic influences, mainly due to the increasing human population density in coastal areas, and are described as “critical transition zones” because of their position at terrestrial, freshwater, and marine interfaces (Levin et al. 2001). Therefore, in any evaluation of the ecological consequences of human activities, such as urbanization and tourism, on the functioning of coastal ecosystems, it is essential to determine the basic structural properties of phytoplankton assemblages in these marine areas (Bosak et al. 2012). The reason is that they play a central role in the structure and functioning of freshwater and marine ecosystems (Pourafrasyabi and Ramezanpour 2014). Phytoplankton populations are well known to be influenced by space-time variations in hydrochemical and physical parameters (Cloern et al. 1989), such as light, temperature, salinity, pH, nutrients, and turbulence (Leterme et al. 2006).

Variations in pH can affect algal growth in a number of ways. It can change the distribution of carbon dioxide species and carbon availability, alter the availability of trace metals and essential nutrients, and at extreme pH levels potentially cause direct physiological effects (Chen and Durbin 1994). In general, changes in pH levels in marine systems appear to correlate with changes in temperature, dissolved oxygen, and phytoplankton production. Conditions of high pH, high phytoplankton production, and low oxygen conditions are characteristic of nutrient-enriched systems and often are found in the coastal waters (Hinga 2002). However, high pH levels are commonly seen in the late afternoon of sunny summers after the consumption of the CO_2_ by photosynthesis process. After sunset, the pH level may significantly be declined due to ending the photosynthesis process (Ghobrani et al. 2014. In the present study, the highest pH value was recorded during summer and a negative correlation was found between the pH values and phytoplankton (*r* = −0.63) and dissolved oxygen (*r* = −0.67).

Oxygen concentration is an index of the balance between processes of food production and food consumption. This balance is a key descriptor of the changing status of the ecosystem. When the balance is disrupted, the oxygen concentration can fall to low levels (Kemker 2013). Accordingly, the study area is well balanced due to the recorded moderate to high values of oxygen. These values synergized with that of phytoplankton (*r* = 0.51). Generally, the slight increase in dissolved oxygen during winter and spring may be due to the increase of oxygen solubility (Nassar 1994). However, the oxygen concentration in the Red Sea is relatively low because of its high salinity and high temperature characteristic of the area (Nassar 2007a). This is also confirmed by Gab-Alla (2007) who reported that Hammam Pharaon hot springs of eastern Suez Gulf (St. 6 in the present study) was slightly acidic (pH 6.3–7.6) and hot (temperature of 25–66 °C) with low oxygen content (0.2–5.5 mg/L) and high salinity (43 %_o_).

The nutrient enrichment of coastal waters is generally the main factor driving the succession and composition of phytoplankton communities (Leterme et al. 2014). Phosphorus availability can impact primary production rates in the ocean as well as species distribution and ecosystem structure and in some marine and estuarine environments; P availability is considered the proximal macronutrient that limits primary production (Paytan and McLaughlin 2007). Generally, the study area exhibited low phosphate concentrations typical of oligotrophic areas (Taş 2013), except the high concentration during autumn at St. 9 (0.30 μM), which may be due to the effect of sewage and oil effluents. However, compared with the water of the Red Sea proper, the Gulf of Suez has very little phosphate and the Red Sea itself is depleted in phosphate as compared to the Gulf of Aden (El-Naggar et al. 2002). In the present study, only diatoms was positively correlated to dissolved phosphate (*r* = 0.69), whereas it was not a limiting factor for other groups.

The inorganic nitrogen pollution in aquatic ecosystems may stimulate the development, maintenance, and proliferation of primary producers resulting in eutrophication of aquatic ecosystems. The Cyanophyceae, Dinophyceae, and Bacillariophyceae appeared to be the major groups that may be stimulated by inorganic nitrogen pollution (Camargo and Alonso 2006).

In general, the high nitrate concentrations enhance the phytoplankton growth during the study period. This was confirmed by the positive correlation between NO_3_ and the total counts of phytoplankton (*r* = 0.66) and the regression analysis (phytoplankton counts = 1379.2341 + 0.662 NO_3_ – 0.33 pH). However, natural phytoplankton communities typically prefer to take up nitrogen in the reduced form of ammonium rather than the oxidized forms nitrite and nitrate. In the present study, there was no correlation between total phytoplankton abundance and ammonium, whereas there was a negative correlation between diatoms and ammonium (*r* = −0.46). This may be due to that various phytoplankton groups and taxa exhibit differential abilities to take up and assimilate dissolved organic nitrogen vs. dissolved inorganic nitrogen (Twomey et al. 2005). According to the low values of dissolved phosphate (0.025–0.3 μM), nitrate (0.18–1.26 μM), and ammonium (0.81–5.36 μM) during 2012–2013, the eastern coast of Suez Gulf is still healthy, relatively unpolluted, and oligotrophic area. This is established with the data reported by Fahmy (2003) who concluded that nitrogen, phosphorus, and reactive silicate concentrations were generally low and allowed classifying the Egyptian Red Sea coastal water as oligotrophic to mesotrophic. This is in addition to the relatively low total abundance of phytoplankton (average of 2989 unit/L), compared with the data reported in its western coast in 1995 (average of 5862 unit/L) by Nassar (2000) and in 2006 (average 15,591 unit/L) by Nassar (2007a), as well as the data reported in 2002 by Shams El-Din et al. (2005) along the both sides of Suez Gulf (average of 6284 unit/L). However, Ghobrani et al. (2014)) mentioned that oligotrophic waters are characterized by high clarity and little counts of algae.

However, the relative high abundance of phytoplankton in this study was found at St. 9 followed by St. 10 with total counts of 4169 and 3840 unit/L, respectively. This may be due to their subject to fractions of petroleum hydrocarbons and sewage discharge, which could promote the phytoplankton growth as reported by Nayar et al. 2005 and Nassar et al. 2014. On the other hand, the lowest occurrence of phytoplankton was recorded at St. 1 and St. 3, with similar total counts of 2218 and 2251 unit/L, respectively. This may be due to the effect of thermal waters discharged from the cooling systems of the Electrical Power Station of Ayon Mousa near St. 1, as well as the high tourist and human activities at the beach of Ras Sudr near St. 3.

As all marine coastal areas, diatoms were the dominant group forming high percentage (67.48 %) and prevailed during the four seasons (53.03–82 %) and at all stations (57–80 %). Whereas, the cyanophytes were the second dominant group, indicating the presence of freshwater discharge in the study area. On the other hand, the contribution of three groups Cyanophyceae, Dinogflagellates, and Chlorophyceae increased during summer at high temperature (28.1–31.5 °C). Eker and Kideyş (2000) suggest that there is a positive relationship between dinoflagellates and water temperature; thus, dinoflagellates may be better adapted to the high temperatures. Most dinoflagellates are found in temperate waters, are most prevalent in summer months (Taylor 1987), and dominate the phytoplankton in warm seasons (Tait 1981). In this connection, Schabhüttl et al. (2012) reported that green algae and diatoms showed a trend to perform better at lower temperatures, while Cyanobacteria showed stronger responses with increasing temperatures in mixed communities. In the present study, temperature was negatively correlated with diatoms (*r* = −0.51) and was positively correlated with chlorophytes (*r* = 0.38), whereas it was not a limiting factor for dinoflagellates and cyanophytes.

However, the dominant species during this study were *C. lorenzianus*, *G. attenuatum*, *P. calcar-avis* in addition to the green alga *P. trabecula*, which appeared at all stations and during all seasons, except winter, indicating the freshwater discharge. Moreover, there were dominant potentially harmful algae, and they appeared frequently and were *P. alata* var. *gracillima* (Özman-Say and Balkis 2012), *P. pungens* (IOC, Casteleyn et al. 2008), and the two cyanophytes *T. erythraeum* and *P. limnetica* (Ramos et al. 2005). Whereas, other potential harmful algae appeared but less frequently or occasionally, such as *Chaetoceos* spp. (Malone 2007), *Cyclotella meneghiana*, *Cylindrotheca closterium*, *Leptocylindrus minimus*, *Skeletonema costatum* (Ismael 2014), *N. longissima*, *Odentella auriata*, *Thalassionema nitzschioides*, *Ceratium fusus*, *Ceratium furca* (Özman-Say and Balkis 2012), *Ceratium tripos* (Ignatiades and Gotsis-Skretas 2010), and *Prorocentrum micans* (Tilstone et al. 2010). The effect of these species is different, as water coloration and foam or mucilage production (Méndez and Ferrari 2002), clogging the fish gills (Malone 2007), secreting domoic acid (Ignatiades and Gotsis-Skretas 2010), anatoxin, mycrocystins (Ramos et al. 2005), or unknown toxins (Ignatiades and Gotsis-Skretas 2010). Although the total count of these species did not exceed 100 cells/L, they are considered dangerous as they can flourish at favorable conditions and they can potentially threat the marine ecosystem (Van Dolah 2000). Thus, the effect of the environmental conditions on the potential harmful dominant species was investigated. The correlation coefficient between the dominant species and the nutrients revealed that *C. lorenzianus* was positively correlated between phosphate (*r* = 0.42) and nitrate (*r* = 0.50) and negatively correlated with ammonium (*r* = −0.51), whereas *G. attenuatum* was positively correlated with nitrate (*r* = 0.49). The diatomate species *P. calcar-avis* was positively correlated with nitrate (*r* = 0.40) and negatively correlated with ammonium (*r* = −0.49), and *P. pungens* was negatively correlated with temperature and pH (*r* = −0.45 and −0.43), respectively, and positively correlated with phosphate and nitrate (*r* = 0.63 and *r* = 0.52), respectively. However, the cyanophyte *P. limnetica* was influenced only by temperature (*r* = 0.50). On the other hand, *P. trabecula*, *P.* var. *gracillima*, and *T. erythraeum* were not affected by any of these physicochemical parameters.

Marine systems are highly dynamic, with biodiversity changing at seasonal and inter-decadal timescales (Nicholas et al. 2010). The relationship between diversity and productivity has been an object of extensive research for both terrestrial and aquatic ecosystems, and the global diversity patterns observed for marine phytoplankton show a unimodal relationship with productivity using phytoplankton biomass as surrogate (Irigoien et al. 2004). High diversity leads to greater community stability and productivity and makes the system less susceptible to invasions Tilman (1999). In this trend, Friedly (2001)) found that diversity was positively related to ecosystem stability, whereas unstable ecosystem will be more likely losses diversity. Meanwhile, Whilm and Doris (1966) mentioned that diversity less than 1 indicates instability or heavy pollution, whereas value exceeding 3 indicates stability or clean water. Accordingly, the stations of study area can be considered stable, where all values were closed to 3 or >3, except St. 7 during summer (2.35), which may be attributed to the low number of the recorded species (19 spp.) and the dominance of only three species: *P. trabecula* (37.79 %), *P. alata* var. *gracillima* (11.34 %), and *P. limnetica* (6.73 %).

The similarity index based on spatial and temporal fluctuations of phytoplankton counts revealed five clusters: cluster 1 (St. 1, 3, and 12), cluster 2 (St. 10), cluster 3 (St. 4, 6, and 9), cluster 4 (St. 2, 8, 5, 7, and 11). The lowest similarity level was 41.11 % between St. 1 and St. 10, whereas the highest level was 76.91 % between St. 3 and St. 12. However, cluster 2, which includes only one station, reflects the unique ecological conditions at this station (oil effluents from Petropil Company). In contrast, the other clusters indicated that the included stations in each cluster have more or less similar ecological conditions, depending on the degree of similarity.

## Conclusion

The study area is considered as oligotrophic and healthy, despite land-base, human, and tourism activities, since the total counts of phytoplankton was low accompanied with low nutrient concentrations and high values of diversity. But due to the appearance of potentially harmful algae species even in low counts, make the region of the eastern coast susceptible to drastic effects at flourishing of these species during favorable conditions. Thus, monitoring continuously of this area is imperative, to follow probable bloom of these species, to predict negative effects resulting from increasing land-based activities in order to protect the eastern coast of Suez Gulf from any undesirable change there.
